# Elevated Microparticles, Thrombin-antithrombin and VEGF Levels in Colorectal Cancer Patients Undergoing Chemotherapy

**DOI:** 10.1007/s12253-020-00854-8

**Published:** 2020-06-24

**Authors:** Marek Z. Wojtukiewicz, Marta Mysliwiec, Ewa Sierko, Monika Sobierska, Joanna Kruszewska, Alina Lipska, Piotr Radziwon, Stephanie C. Tucker, Kenneth V. Honn

**Affiliations:** 1 Department of Oncology, Medical University of Bialystok, 12 Ogrodowa St, 15-027 Bialystok, Poland; 2 Department of Radiotherapy, Comprehensive Cancer Center, Bialystok, Poland; 3 Regional Centre for Transfusion Medicine, Bialystok, Poland; 4 Department of Hematology, Medical University of Bialystok, Bialystok, Poland; 5 Bioactive Lipids Research Program, Department of Pathology-School of Medicine, Wayne State University, Detroit, MI 48202 USA; 6 Karmanos Cancer Institute, 48201 Detroit, MI USA; 7 Department of Chemistry, Wayne State University, 48202 Detroit, MI USA; 8 Department of Oncology, Wayne State University, 48202 Detroit, MI USA

**Keywords:** Endothelium-derived microparticles, Blood coagulation, Angiogenesis, Colorectal cancer

## Abstract

Hypercoagulable state and neoangiogenesis are common phenomena associated with malignancy. Cancer patients have increased levels of circulating endothelium-derived microparticles (EMPs), which have been hypothesized to be involved in numerous pathophysiological processes. Hemostasis and angiogenesis are also activated in colorectal cancer (CRC) patients. The study aimed to investigate potential influence of chemotherapy on EMPs, thrombin anti-thrombin complex (TAT) and vascular endothelial growth factor (VEGF) levels in CRC patients undergoing chemotherapy. The study group consisted of 18 CRC patients: 8 stage III colon cancer (CC) and 10 stage IV rectal cancer (RC) patients. EMPs, TAT and VEGF levels were assessed before chemotherapy and after the third course. Results were compared with 10 healthy subjects. EMP concentration was measured by flow cytometry, while TAT and VEGF concentrations were assayed employing ELISA. Compared to the control group, CC and RC patients had significantly higher levels of tissue factor (TF)-bearing and non-TF-bearing EMPs before and after three courses of chemotherapy. VEGF concentrations in CRC patients were higher than in the control groups and increased following chemotherapy. TAT levels were elevated in CRC patients before chemotherapy compared to healthy subjects and significantly increased after the third course of chemotherapy. No significant correlation was found either between EMP and TAT levels, or between EMP concentrations and VEGF levels in the study group. CRC patients have increased EMPs, and TAT as well as VEGF levels tend to increase during chemotherapy.

## Background

Close association between malignancy and blood coagulation disorders has been recognized for over 130 years [1]. Cancer is one of the major independent risk factors for development of venous thromboembolic disease (VTE) [2, 3], which is the second most frequent reason for mortality in cancer patients [4, 5]. Indeed, cancer patients bear significantly higher (4–7-fold) risk of VTE than their healthy counterparts [6]. The risk of VTE is also increased in colorectal cancer (CRC) patients [7]. Furthermore, oncological therapeutic procedures additionally elevate the risk of thrombosis. Chemotherapy, for example, has been linked to 4.5 to 6-fold incremented risk of VTE [8]. In fact, coagulopathy and angiogenesis are among the most consistent host reactions associated with malignancy.

Most primary tumors and metastatic lesions cannot grow beyond 2 to 3 mm^3^ in size in the absence of vascularization [9]. Therefore, angiogenesis, a process of new blood vessel formation, is necessary for growth of different types of cancer, including colorectal cancer. There are multiple interactions between coagulation factors of the hemostatic system and angiogenic activity in cancer patients. Various components of the hemostatic system contribute to either promotion or inhibition of angiogenesis and may change a net precarious proangiogenic/antiangiogenic balance [10]. Tissue factor (TF), which is the main procoagulant, can contribute to tumor angiogenesis via mechanisms that are either dependent on or independent of blood clotting activation (e.g., TF-signaling elevating expression of molecules such as the main proangiogenic factor - vascular endothelial growth factor, VEGF). The procoagulant activity of TF leads to thrombin generation, platelet activation and fibrin formation, all of which influence tumor angiogenesis [11–13]. The expression of both TF [14] and VEGF [15] is controlled by hypoxia. Elevated level of VEGF is a poor prognostic factor in many solid tumors [16].

It has been proven that the level of endothelium-derived microparticles (EMPs) is significantly higher in cancer patients, thereby contributing to blood coagulation activation in this group of patients [17, 18]. Negatively charged phospholipids expressed on MP are considered to be the main initiators of the coagulation cascade [19]. Additionally, it has been shown that sphingosine 1-phosphate (S1P) strongly potentiates thrombin-induced TF expression in endothelial cells, suggesting its role in blood coagulation. A role for S1P has also been demonstrated in the process of angiogenesis [20], thus explaining why exposed procoagulant phospholipids and specific receptors at the surface of MPs act as biomessengers, linking coagulation and angiogenesis. Endothelium-derived microparticles represent about 10–15% of the total MPs in plasma of healthy subjects and they can be quantified by flow cytometry using two panels of monoclonal antibodies [21]. EMPs originating from apoptosis are characterized primarily by surface antigen CD31, while EMPs derived from cell activation exhibit increased expression of CD62E antigen [22].

Colorectal cancer patients are known to have higher levels of circulating MPs than healthy controls [23]. Moreover, the population of MPs in CRC patients is highly heterogeneous and some subpopulations may impact blood coagulation and tumor angiogenesis in these patients [24, 25].

The aim of this study was to determine EMP, TAT and VEGF levels in colon cancer patients undergoing adjuvant chemotherapy and in rectal cancer patients undergoing palliative chemotherapy.

## Methods

### Study Population

Blood samples were obtained from eighteen patients (10 males and 8 females) with histopathology-diagnosed G2 adenocarcinoma of colon or rectum. Patients’ ages ranged from 41 to 73 years. Depending on the type of chemotherapy, patients were divided into two subgroups. The first subgroup consisted of stage III colon cancer (CC) patients: 4 males and 4 females (aged 44–73) who underwent adjuvant chemotherapy (AC) after surgery. The second subgroup included stage IV rectal cancer (RC) patients with distant metastases: 6 males and 4 females (aged 41–72) who underwent palliative chemotherapy (PC).

After surgery, CC patients received AC according to the FOLFOX regimen. Rectal cancer patients were given first line PC according to the CLF_1_ regimen, which was a standard therapy in Poland at the time. Targeted therapy was not available at that time in routine clinical settings. Control blood samples were obtained from healthy subjects: 3 males and 7 females aged 40–70 years.

### EMP Enumeration by Flow Cytometry

The blood was drawn twice from each patient in the study group: before chemotherapy and after the 3rd course of the treatment. In the control group, blood samples were collected once, under the same conditions as in the study group.

Routinely, 5 ml of blood were collected from the ulnar vein from all patients using a 21gauge needle into S-Monovette sample tubes (Sarstedt) containing anticoagulant (0.129 mol/L sodium citrate). Two steps of centrifugation were performed in order to isolate microparticles from whole blood. First, blood samples were centrifuged at 5000 x g at 24 °C for 5 min without centrifuge brake (removal of blood cells) to prepare platelet-free plasma (PFP). To collect MP, 600 µL of PFP were centrifuged at 14000 x g at 20 °C for 5 min and the supernatant was removed. The MP pellet was then resuspended in 500 µL of diluted Annexin V FITC binding buffer (BD Bioscience, Franklin Lakes, NJ, USA). The storage time of samples never exceeded 30 min following venipuncture, including centrifugation steps.

Quantification of pelleted MP numbers and their phenotypic characterization were conducted by flow cytometry (FACSCalibur, Becton Dickinson, USA). MP size gate was set between 200 and 1000 nm using fluorescent latex beads 1 µm (Precision Size Standards, Polysciences). The total amount of MP was defined as all events falling within the MP gate. To count the numbers of MP, an aliquot of suspended MP pellet (100 µL) was added to TruCount (Becton Dickinson, USA) (100 µL), followed by counting up to 2000 of the 0.5 µL bead component of TruCount (total absolute count of MP = (events in region except beads/events in region of beads) x (absolute number of beads/µL/sample volume[µL]).

**Fig. 1:**
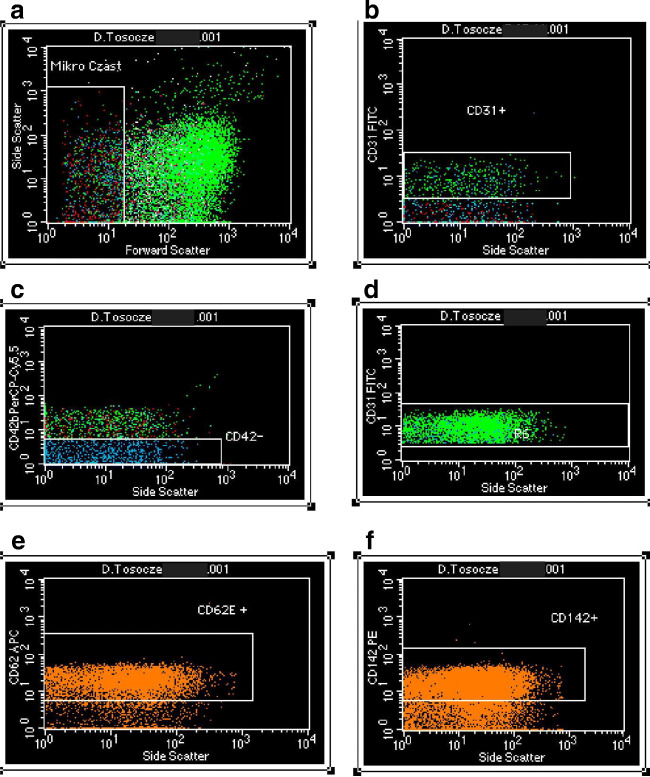
Representative dot plots from the FACSCalibur flow cytometer. Patient’s sample. Forward and side scatter of isolated microparticles (MPs). (**A**) Gated population of all MPs; stained with annexin-V and using TruCOUNT internal calibrator. The forward scatter cut off was set using 1.0-µm standard beads to define the upper limit of MPs population; (**B**) the MPs gate for Fluorescein isothiocyanate (FITC)-conjugated anti-CD31+; (**C**) the gate for MPs negative for Peridinin-chlorophyll protein-Cy5.5 (PerCP-Cy5.5)-conjugated anti-CD42+; (**D**) gate (R6) for MPs positive for CD31 + and negative for CD42+; (**E**) the MPs gate for Allophycocyanin (APC)-conjugated anti-62E+; (**F**) the MPs gate for Phycoerythrin (PE)-conjugated anti-142+

The determination of TF-negative EMPs (TF^−^EMPs) and TF-positive EMPs (TF^+^EMPs) was performed employing combination of antibodies: CD31^+^/ CD62E^+^/CD142^−^/CD42b^−^ and CD31^+^/ CD62E^+^/CD142^+^/CD42b^−^, respectively, using a BD FACSCalibur flow cytometer (USA). monoclonal antibody anti-CD42b was used to exclude platelet- and megakaryocyte-derived MPs, whereas anti-CD142 was required for TF measurement (Fig. 1).

All monoclonal antibodies used in the study were purchased from Becton Dickinson Pharmingen (San Diego, CA, USA): CD31 stained with FITC, CD62E stained with APC, PE-Cy5 labeled anti-human CD42b, PE labeled anti-human CD142 and kappa isotype control: FITC labeled mouse IgG1, APC labeled mouse IgG, PE-Cy5 labeled mouse IgG1 50, PE labeled mouse IgG1. The remaining reagents were manufactured by Becton Dickinson Bioscience (San Jose, CA, USA) and included: CellWASH, FACS Lysing Solution, PBS Wash buffer, FACS Flow Sheath Fluid, FASC Lysing Solution.

### Vascular Endothelial Growth Factor (VEGF) and Thrombin-antithrombin Complex (TAT) Measurement by ELISA

Blood was collected under the same conditions as described in the method of EMP enumeration by flow cytometry. The number of plasma samples was the same.

The samples were centrifuged at 1500 x g for 15 min at room temperature in order to yield plasma. Sample storage time before centrifugation never exceeded 30 min following venipuncture. The plasma was frozen and stored at -20^0^C until analysis. Before the assay, all samples were slowly thawed and gently vortexed by hand to resuspend. VEGF and thrombin-antithrombin complex (TAT) plasma levels were determined using an immunoenzymatic method.

To assess TAT and VEGF concentrations, IMUBIND TAT (American Diagnostica, USA) and VEGF Quantikine ELISA (R&D Systems, USA) kits were used, respectively.

Correlations between plasma EMP levels and either plasma TAT concentration or plasma VEGF concentration were analyzed.

### Statistical Analysis

Data were expressed as median, mean and standard deviation. The Shapiro-Wilk test was used to check for normal distribution of the results. To compare the levels of EMPs, TAT and VEGF between groups, the Wilcoxon Signed Rank test for dependent data was used. Correlations between EMP and TAT as well as between EMP and VEGF were assessed with the Spearman test. Statistical significance was defined by p < 0.05. Analyses were performed with STATISTICA 10 software and Microsoft Excel 2010.

## Results

### Concentration of TF^−^EMPs in Plasma of Colon Cancer Patients Undergoing Adjuvant Chemotherapy and in Plasma of Rectal Cancer Patients Receiving Palliative Chemotherapy

TF^−^EMPs were characterized by phenotype CD31^+^CD142^−^CD42b^−^. Colon cancer patients had statistically significantly higher concentrations of TF^−^EMPs before treatment (1640 ± 648/µl (p < 0.05) and after the 3rd cycle of adjuvant chemotherapy (1940 ± 294/µl (p < 0.05) in comparison to the control group (834 ± 667/µl) (Table 1).

**Table 1:** Concentrations of tissue factor-negative endothelial microparticles (TF^−^EMPs) and tissue factor-positive endothelial microparticles (TF^+^EMPs) in the plasma of colon cancer patients undergoing adjuvant chemotherapy and in the control group

Time of assessment	EMP CD31^+^/ CD62E+/CD142^−^/CD42b^−^ [quantity/µl]		EMP CD31^+^/CD62E+/CD142^+^/ CD42b^−^ [quantity/µl]	
	x ± SD	Me	x ± SD	Me
control group	834 ± 667	1141	688 ± 647	345
before adjuvant chemotherapy	1640* ± 648	1451	1655* ± 882	1495
after 3 courses of adjuvant chemotherapy	1940* ± 294	2065	1149* ± 550	1348

CD31, CD62E – EMP surface antigens

CD42b – platelets and megakaryocyte surface antigens

x –mean

SD –standard deviation

Me – median

* - significant difference in comparison with the control group

TF^−^EMP concentrations in RC patients were significantly higher in comparison with the control group before PC (1646 ± 884/µl vs. 834 ± 667/µl, p < 0.05), as well as after the 3rd course of chemotherapy (2087 ± 324/µl, p < 0.05) (Table 2).

**Table 2:** Concentrations of tissue factor-negative endothelial microparticles (TF^−^EMPs) and tissue factor-positive endothelial microparticles (TF^+^EMPs) in the plasma of rectal cancer patients undergoing palliative chemotherapy and in the control group

Time of assessment	EMP CD31^+^/ CD62E+/CD142^−^/CD42b^−^ [quantity/µl]		EMP CD31^+^/CD62E+/CD142^+^/ CD42b^−^ [quantity/µl]	
	x ± SD	Me	x ± SD	Me
control group	834 ± 667	1141	688 ± 647	345
before palliative chemotherapy	1646* ± 884	1771	1417* ± 643	1389
after 3 courses of palliative chemotherapy	2087* ± 324	2023	1613* ± 231	1622

CD31, CD62E – EMP surface antigens

CD42b – platelets and megakaryocyte surface antigens

x –mean

SD –standard deviation

Me – median

*- significant difference in comparison with the control group

### Concentration of TF^+^EMPs in Colon Cancer Patients Undergoing Adjuvant Chemotherapy and in Rectal Cancer Patients Receiving Palliative Chemotherapy

The TF^+^EMPs were the vesicles with phenotype CD31^+^CD62E^+^CD142^+^CD42b^−^. Colon cancer patients had significantly higher TF^+^EMP concentrations before treatment (1655 ± 882/µl, p < 0.05) and after adjuvant chemotherapy (1149 ± 550/µl, p < 0.05) when compared to the control group (688 ± 647/µl). There was no statistically significant difference in TF^+^EMPs concentrations in colon cancer patients before and after adjuvant chemotherapy (Table 1).

Compared to healthy subjects (688 ± 647/µl), TF^+^EMP levels in rectal cancer patients were significantly higher before treatment (1417 ± 643/µl, p < 0.05) and after palliative chemotherapy (1613 ± 231/µl, p < 0.05). There was no statistically significant difference in TF^+^EMP concentrations with the cohort of rectal cancer patients before and after palliative chemotherapy (Table 2).

### Concentration of VEGF in Colon Cancer Patients Undergoing Adjuvant Chemotherapy and Rectal Cancer Patients Receiving Palliative Chemotherapy

**Fig. 2:**
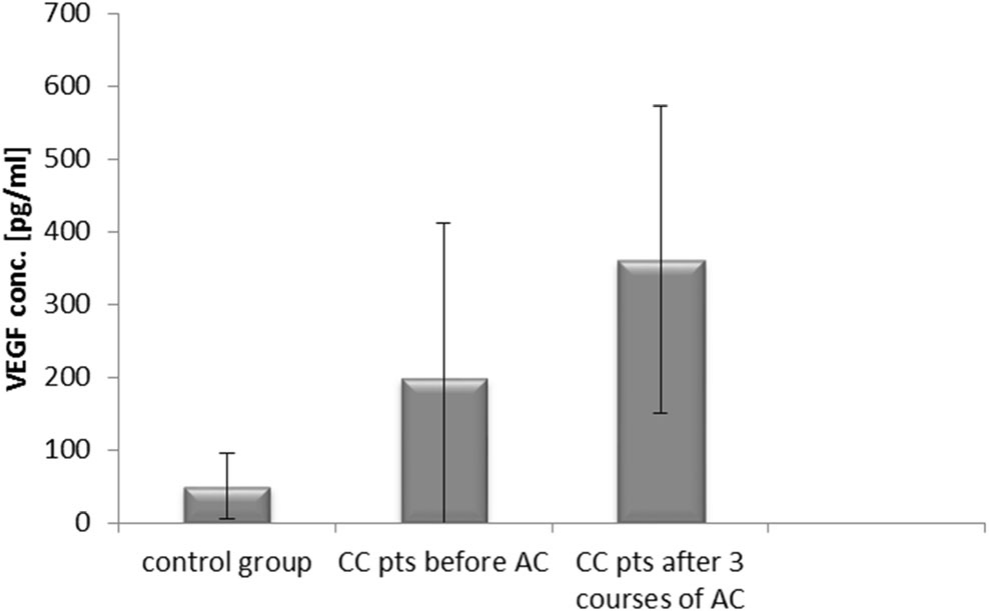
Vascular endothelial growth factor (VEGF) concentrations in colon cancer (CC) patients (pts) undergoing adjuvant chemotherapy (AC) and in the control group

According to the analysis, VEGF concentrations in colon cancer patients were statistically significantly higher before treatment (199.9 ± 211.9 pg/ml, p < 0.05) and after the 3rd course of adjuvant chemotherapy (361.5 ± 210.5 pg/ml, p < 0.05) when compared to the control group (50.2 ± 44.4 pg/ml). Patients after adjuvant chemotherapy had significantly higher VEGF levels compared to baseline values (p < 0.05) (Fig. 2).

**Fig. 3:**
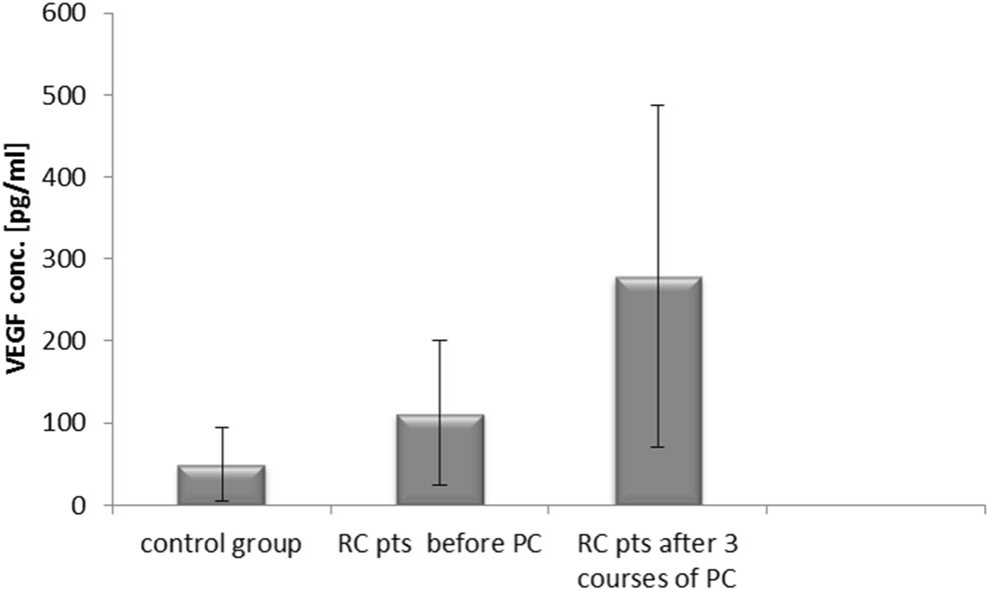
Vascular endothelial growth factor (VEGF) concentrations in rectal cancer (RC) patients (pts) undergoing palliative chemotherapy (PC) and in the control group

Compared to the control group (50.2 ± 44.4 pg/ml), rectal cancer patients had significantly higher (about 2-fold) VEGF levels before palliative chemotherapy (112.5 ± 88.6 pg/ml, p < 0.05) and after palliative chemotherapy (278.8 ± 209 pg/ml, p < 0.05) (Fig. 3).

### Concentration of TAT in Colon Cancer Patients Undergoing Adjuvant Chemotherapy and Rectal Cancer Patients Receiving Palliative Chemotherapy

**Fig. 4:**
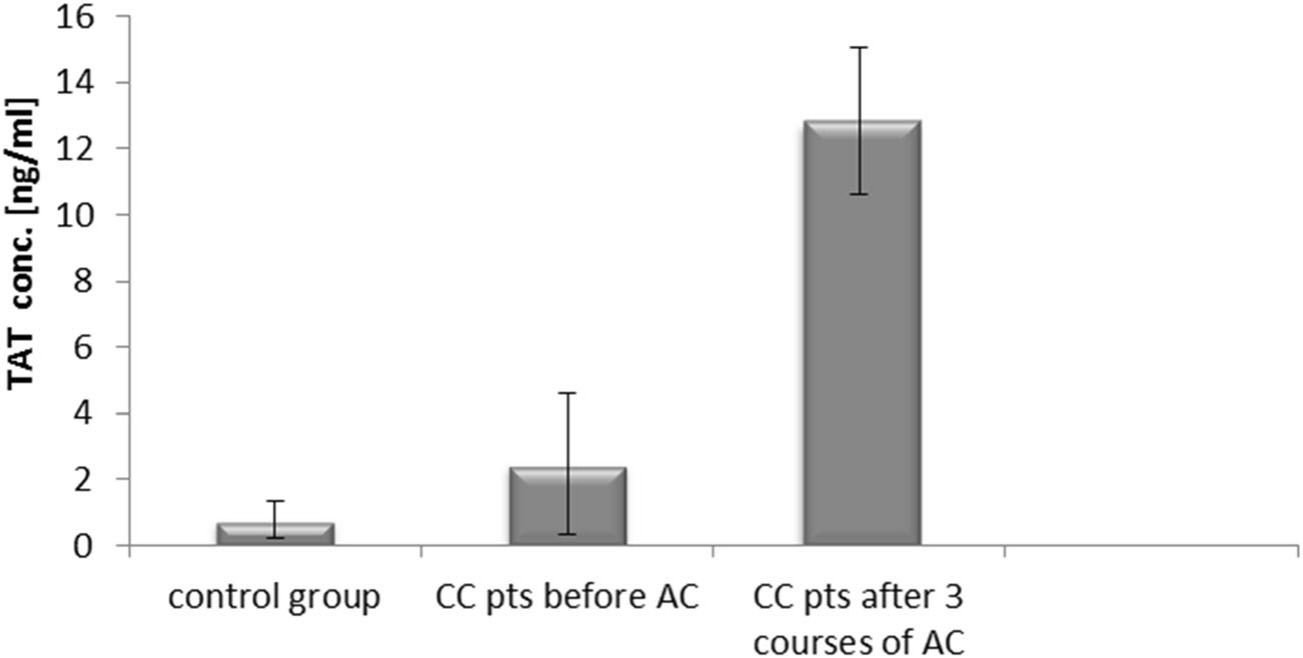
Thrombin-antithrombin complex (TAT) concentrations in colon cancer (CC) patients (pts) undergoing adjuvant chemotherapy (AC) and in the control group

The mean concentration of TAT in colon cancer patients before treatment (2.46 ± 2.15 ng/ml) was significantly higher when compared to the control group 0.77 ± 0.57 ng/ml, p < 0.05) (Fig. 4).

After 3rd course of adjuvant chemotherapy, TAT levels (12.83 ± 2.22 ng/ml) were significantly higher when compared to both pre-chemotherapy concentrations (2.46 ± 2.15 ng/ml, p < 0.05) and the control group (0.77 ± 0.57 ng/ml, p < 0.05) (Fig. 4).

**Fig. 5:**
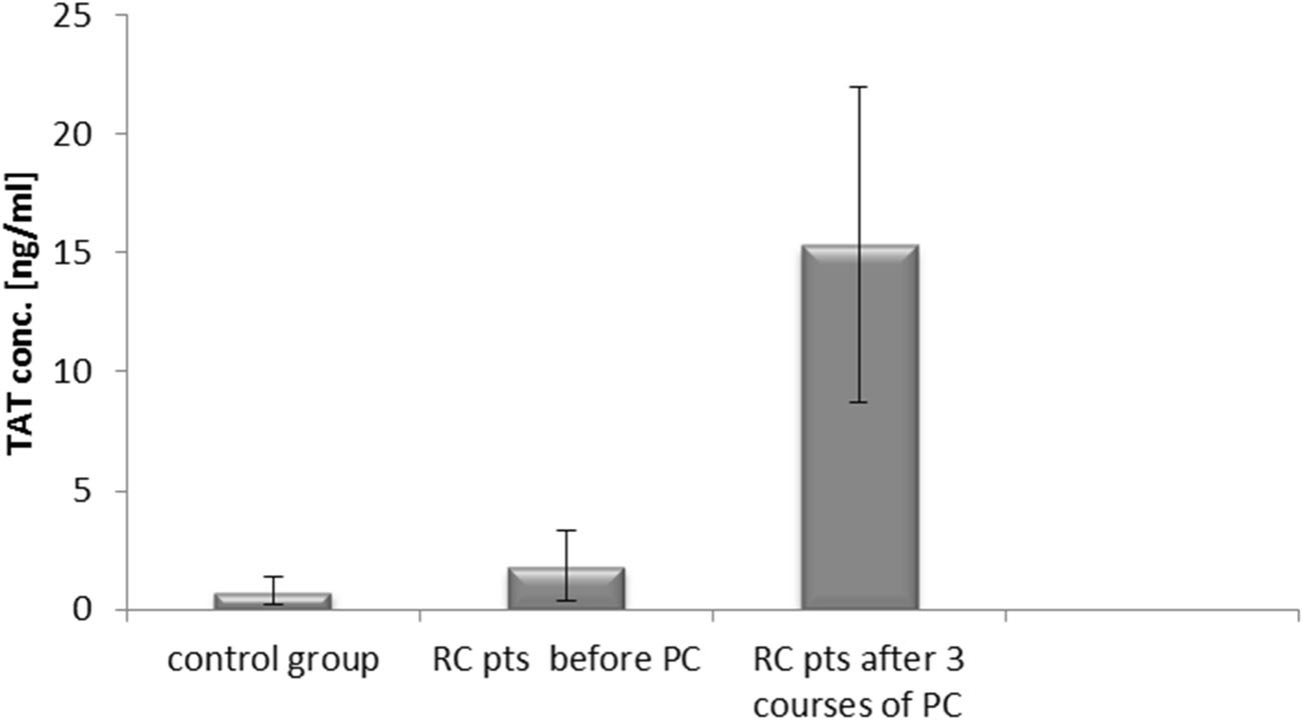
Thrombin-antithrombin complex (TAT) concentrations in rectal cancer (RC) patients (pts) undergoing palliative chemotherapy (PC) and in the control group

The mean TAT concentration in rectal cancer patients before treatment was significantly higher than in healthy subjects (1.82 ± 1.49 ng/ml vs. 0.77 ± 0.57 ng/ml, p < 0.05). Rectal cancer patients had significantly higher TAT levels after the 3rd course of palliative chemotherapy (15.3 ± 6.63 ng/ml) compared to baseline values (1.82 ± 1.49 ng/ml, p < 0.05) and the control group (0.77 ± 0.57 ng/ml, p < 0.05) (Fig. 5).

### Correlation Between the Levels of EMPs and TAT as well as Between EMPs and VEGF Levels in Colon Cancer and Rectal Cancer Patients Undergoing Adjuvant Chemotherapy and Palliative Chemotherapy

There was no statistically significant correlation between TF^+^EMPs and TAT levels in CC patients before treatment (r=-0.626, p = 0.096) and after AC (r = 0.047, p = 0.91). No significant correlation was demonstrated also between TF^+^EMPs and TAT concentrations in rectal cancer patients before treatment (r=-0.309, p = 0.881) and after palliative chemotherapy (r=-0.058, p = 0.881). Similarly, no correlation was found between TF^−^EMPs and VEGF concentrations in colon cancer patients before treatment (r=-0.108, p = 0.798) and after adjuvant chemotherapy (r = 0.238, p = 0.57). However, a significant negative correlation was demonstrated between TF^+^EMPs and VEGF levels before treatment (r = 0.266, p = 0.487) and after palliative chemotherapy (r=-0.811, p = 0.007).

## Discussion

A number of abnormalities in blood coagulation in association with cancer have been demonstrated, including elevated fibrinogen, thrombin generation, enhanced expression of TF and cancer procoagulant [26]. An imbalance between activators and inhibitors of the hemostatic system is observed, which may contribute not only to increased prothrombotic tendency but also to tumor invasion, progression and metastasis [27–32]. A growing body of evidence indicates that different forms of anticancer treatment affect the hemostatic system in cancer patients [8, 33–36].

Circulating MPs contribute to inflammatory responses, vascular remodeling, angiogenesis and apoptosis, and are involved in atherothrombosis [37–39]. Increased levels of circulating MPs have been reported in a wide range of diseases connected with thrombotic risk. Cancer patients appear to have elevated levels of procoagulant MPs compared to healthy controls [40–42]. Cancer-related hypercoagulability may in part result from elevated levels of circulating microparticle-bound TF (MPTF). Among several subgroups of MPs, platelet microparticles (PMPs) and EMPs may be of special importance [20, 43].

EMPs may offer a potentially unique set of biomarkers of thrombotic disorders, including cancer-related thrombosis [40, 42, 44]. In fact, the assessment of EMP levels has been receiving increased attention as a possible diagnostic/prognostic indicator for thromboembolism [45]. Chirinos et al. [46] reported significantly increased concentrations of EMPs during acute VTE. Another study revealed that the levels of circulating platelet microparticle-associated TF (PMPTF) and EMP-associated TF (EMPTF) were significantly increased in comparison to other MPTF in recurrent deep venous thrombosis (DVT) [47]. Cancer patient MPTF levels and associated activities were analyzed by several groups of investigators. Tesselaar et al. [44] found increased levels of TF-positive MPs in pancreatic and breast adenocarcinoma patients compared with controls. Hron et al. [40] reported a two-fold higher level of TF-positive MPs in the plasma of advanced CRC compared to healthy subjects. Khorana et al. [41] described that the activity of TF-positive MPs may be predictive of VTE in pancreatic cancer patients. Thaler et al. [42] investigated the predictive value of MPTF activity in terms of mortality and VTE occurrence in pancreatic, gastric, colorectal and brain cancer patients. MPTF activity was not associated with subsequent VTE incidence in any of the four groups of patients, while a strong association between mortality and MPTF activity was demonstrated exclusively in pancreatic cancer patients.

The current study analyzes EMPs, TAT and VEGF levels in CRC patients undergoing adjuvant or palliative chemotherapy. Thrombin-antithrombin complexes have been used as a well-established marker for thrombin generation [48], where higher TAT levels correspond with enhanced TF expression in pancreatic cancer patients [49]. Increased TAT concentrations were demonstrated in CC patients [50]. In the present study significantly higher concentrations of TAT were observed in CRC patients in comparison to healthy subjects, both before treatment and after the 3rd course of chemotherapy. Elevated TAT in CRC patients treated with adjuvant or palliative chemotherapy might suggest an influence of the treatment on blood coagulation activation. There does not appear to be a significant correlation between EMPs and TAT levels in CRC patients. Our results are in accordance with findings of Tesselaar et al. [51], who found only a weak correlation between MPTF activity and TAT levels in cancer patients. Lechner et al. [52] reported marginal TF expression on EMPs and found that thrombin generation induced by EMPs was independent of TF. The study also suggested that the release of EMPs does not contribute to hemostatic abnormalities in cancer patients.

Vascular endothelial growth factor is the major factor stimulating tumor angiogenesis [53–55]. Expression of VEGF correlates with poor prognosis and metastatic dissemination in a wide variety of human malignancies, e.g., lung [51], gastric [56] and colorectal [57] cancer. It has been suggested that serum VEGF levels could be a prognostic marker in CRC patients [58]. We observed significantly higher VEGF levels in CRC patients when compared to healthy subjects, as well as increased VEGF levels in cancer patients undergoing chemotherapy in comparison to patients before treatment. Adjuvant and palliative chemotherapy may contribute to increased VEGF concentrations in cancer patients, which has been observed by other investigators. Breast cancer patients experienced an increasing trend in plasma VEGF levels during chemotherapy, and the types of treatment regimens may differentially effect circulating VEGF levels [59]. In our study no significant correlation was found between EMPs and free VEGF concentrations in CRC patients undergoing adjuvant or palliative chemotherapy. Other investigators reported a correlation between EMPs and VEGF concentrations in cancer patients treated with VEGF inhibitors [60]. However, our patients underwent chemotherapy without antiangiogenic agents. It is noteworthy that EMPs may stimulate angiogenesis via molecules other than VEGF, e.g. TGF-β, proteolytic enzymes [61], sphingomyelin [62] and micro-RNA [63]. This points to a more complex interplay among various proangiogenic factors at a tumor site, which might not necessarily be reflected by the concentration of free VEGF in the plasma.

## Conclusions

CRC patients exhibit elevated TAT and VEGF levels, which tend to increase during the course of chemotherapy. EMPs may also contribute to the observed changes. However, additional studies performed on a larger group of patients and employing more parameters reflecting hemostasis and angiogenesis are warranted.

## Data Availability

All data generated or analyzed during this study are included in this published article.

## Abbreviations

CC: colon cancer
CRC: colorectal cancer
ELISA: enzyme–linked immunosorbent assay
EMPs: endothelium-derived microparticles
RC: rectal cancer
TAT: thrombin-antithrombin complex
VEGF: vascular endothelial growth factor
VTE: venous thromboembolism
